# Pathogen detection, testing, and control in fresh broccoli sprouts

**DOI:** 10.1186/1475-2891-5-13

**Published:** 2006-04-21

**Authors:** Jed W Fahey, Philippe J Ourisson, Frederick H Degnan

**Affiliations:** 1 Department of Pharmacology and Molecular Sciences, Lewis B. and Dorothy Cullman Cancer Chemoprotection Center, Johns Hopkins University School of Medicine, Baltimore, Maryland, USA; 2 Department of International Health, Center for Human Nutrition, Johns Hopkins University Bloomberg School of Public Health, Baltimore, Maryland, USA; 3 Quality Associates, Inc., Columbia, Maryland, USA; 4 King and Spalding LLP, Washington D.C., USA

## Abstract

**Background:**

The recent increased interest in consuming green vegetable sprouts has been tempered by the fact that fresh sprouts can in some cases be vehicles for food-borne illnesses. They must be grown according to proper conditions of sanitation and handled as a food product rather than as an agricultural commodity. When sprouts are grown in accordance with the criteria proposed from within the sprout industry, developed by regulatory agencies, and adhered to by many sprouters, green sprouts can be produced with very low risk. Contamination may occur when these guidelines are not followed.

**Methods:**

A one year program of microbial hold-and-release testing, conducted in concert with strict seed and facility cleaning procedures by 13 U.S. broccoli sprout growers was evaluated. Microbial contamination tests were performed on 6839 drums of sprouts, equivalent to about 5 million consumer packages of fresh green sprouts.

**Results:**

Only 24 (0.75%) of the 3191 sprout samples gave an initial positive test for *Escherichia coli *O157:H7 or *Salmonella *spp., and when re-tested, 3 drums again tested positive. Composite testing (e.g., pooling up to 7 drums for pathogen testing) was equally sensitive to single drum testing.

**Conclusion:**

By using a "test-and-re-test" protocol, growers were able to minimize crop destruction. By pooling drums for testing, they were also able to reduce testing costs which now represent a substantial portion of the costs associated with sprout growing. The test-and-hold scheme described herein allowed those few batches of contaminated sprouts to be found prior to packaging and shipping. These events were isolated, and only safe sprouts entered the food supply.

## Background

Green sprouts have been a part of the human diet for much of recorded history. Their commercial production has been a small niche industry in the U.S. for the past 30 or so years, but they are much more widely consumed in countries like Japan, where they are part of mainstream diets. In the past 8 years broccoli sprouts have gained increased scientific attention due to their high content of phytochemicals that are involved in protection against cancer and other degenerative diseases [1-7].

Industry experts estimate that about 15 million pounds of fresh green sprouts are now grown in the U.S. annually and the vast majority of these are alfalfa sprouts. Sprouts are grown from seeds placed in environmentally controlled, hydroponic conditions and incubated in warm, moist, nutrient-rich conditions [1.5 m. diameter, slowly rotating "drums" charged with ca. 20 kg of seed], which are ideal environments for microbial growth. If *Escherichia coli *or *Salmonella *spp. are present on the surface of the seed, it is likely that they will multiply in the sprouting environment. To date, no practical methods have been developed to check the growth of these contaminants during germination and sprout growth or processing. They must therefore be prevented from entering the process, or if contamination occurs, affected final product must be identified and destroyed. It is therefore essential that seeds to be used for sprouting, undergo surface-disinfection by treatment with a biocide. The efficacy of such agents, most notably calcium hypochlorite, has been extensively documented in the laboratories of Beuchat and colleagues, [8-12] as well as others [13-15]. When these agents are used correctly, the resulting sprouts are safe to eat. A recommendation to use such a surface disinfection process is now part of a guidance that the FDA issued in 1999 [16].

This recent increased interest in consuming green vegetable sprouts has been tempered by the fact that fresh sprouts can in some cases be vehicles for food-borne illnesses, if not grown according to proper conditions of sanitation and handled as a food product rather than as an agricultural commodity [17-19]. When sprouts are grown in accordance with the criteria proposed from within the sprout industry, developed by the FDA [16], and adhered to by many sprouters, green sprouts can be produced with very low risk. Contamination can occur when these guidelines are not followed [19,20].

Whereas chlorination of seeds dramatically reduces the chances of growing a contaminated product, adherence to good manufacturing practices and integration of Hazard Analysis Critical Control Point (HACCP) plans [21] further reduce such chances. The ultimate control point for microbial safety is the product, thus incidents of contamination, when they occur, can only be ascertained by testing each lot of sprouts prior to releasing them for sale (hold-and-release testing). This is best done by testing the spent irrigation water. The United States FDA has issued a guidance document for growers, with considerable input from the sprouting industry, which is designed to prevent contaminated sprouts from ever reaching the public [16]. The FDA followed up issuance of this guidance with an inspection of 150 sprouters, and determined that only about half of them were complying with the guidance [22]. We have examined a subset of sprouters in whose facilities compliance with the FDA guidance was verified based upon inspections by two third party auditors.

## Methods

### Hold-and-release testing

The growers, 13 producers or co-packers of broccoli (BroccoSprouts^®^) and other sprouts for the company Brassica Protection Products (Baltimore, MD, USA) during the 2001 calendar year, were selected for this review. These growers were selected because they are established companies that grow many different kinds of green sprouts, and they had previously agreed to comply with rigid standards of sanitation. Geographically, they are distributed around the United States with respect to both plant siting and product distribution. In particular, all surveyed growers follow all of the steps specified in the FDA Guidance for Industry [16]. Specifically, they surface-disinfect all seeds and perform microbial testing of the spent irrigation water from each batch of green sprouts produced, and they are subject to announced and unannounced third party audits and state and federal inspections of these procedures.

### Seed treatment and sprout production

Briefly, surface disinfection of seeds is accomplished by exposing seeds to 20,000 ppm of calcium hypochlorite for 15 min, followed by extensive rinsing to remove residual chlorine. Sprouts are grown in trays or drums which are provided with only light (fluorescent, incandescent and/or filtered sunlight), heat (constant temperature), and clean water (filtered, well, or municipal-chlorinated). A sample of the spent irrigation water, typically 1 L, is collected after 48 hours of sprout growth. Since the sprouts typically are grown for 72 – 120 h, a rapid microbial test permits the sprouters to abort contaminated batches of sprouts prior to packaging, or to shipping and distribution. All sprouts were held until test results were obtained. In cases where presumptive positives were obtained, the refrigerated, unused portion of the 48 hour spent irrigation water sample was used for re-testing as specified by the FDA guidance [16]. Typically, the confirmation analysis was performed using a different, more specific method. In at least one situation, the grower was collecting individual samples, and the testing lab was preparing a composite sample for testing. In this case, a confirmation was performed on each individual sample (one water sample per drum).

### Data collection

All growers who contributed data for this study were requested to directly forward their hold-and-release testing results for the calendar year 2001 to Quality Associates Incorporated (QAI; Columbia, MD, USA). Many growers included a description of the green sprouts grown in each batch with these results. In addition to broccoli sprouts, the growers reported growing the following green sprouts: alfalfa, clover, radish, onion, pea, sunflower, and a variety of mixes. Data that was exclusively derived from batches of bean sprouts (e.g. mungbean and soybean) were not used herein. However, in some cases pooled water samples that were tested included water from both green sprouts and bean sprouts, and these test results are included in the analyses.

The raw data utilized were copies of the analytical reports, as they were received by the growers from the contract analytical laboratories that performed the microbial testing. All growers but one used the services of an external microbiological testing laboratory, to which samples could be delivered on the day of collection. One grower had instead set up an in-house laboratory for these analyses and all laboratory notes were provided to us by this grower.

### Microbial testing

The analytical laboratories generally used test kits designed for simple and quick screening of the samples for contamination by specific bacteria. All samples were screened for *Escherichia coli *O157:H7 and for *Salmonella *spp. Most of the testing for *E. coli *O157:H7 (over 75%) was performed using a test kit identified in the FDA Guidance for Industry [16]. Only about 10% of the testing for Salmonella was performed using a test kit identified in this FDA Guidance. Most kits were Association of Official Analytical Chemists (AOAC) Official Methods; one kit was an AOAC Performance Tested Method. Instead of a kit, one laboratory used the official FDA Bacterial Analytical Method [23] for *Salmonella *testing. The methods used by two laboratories (both for *E. coli *and *Salmonella*) and for a third (only for *Salmonella*) were not specified. The specific methods are listed and referenced in Table 1. All results were entered into a database and data entries were independently verified by an auditor at QAI.

**Table 1:** Methods used for hold-and-release testing.

Bacteria	AOAC No.	AOAC Status	Test kit name	Sensitivity (%)	Specificity (%)
*E. coli *0157:H7	996.09^*a*^	Official Method	Biocontrol VIP EHEC for *E.coli *O157:H7	>98	>99
	996.10^*b*^	Official Method	Biocontrol Assurance EIA EHEC	100	>98
	2000.14^*c*^	Official Method	Neogen Reveal *E.coli *O157:H7	>89	>98
*Salmonella *spp.	999.09^*d*^	Official Method	Biocontrol VIP for *Salmonella*	>77	>98
	996.08^*e*^	Official Method	bioMerieux VIDAS SLM	>96	100
	960801^*f*^	Performance Tested Method	Neogen Reveal *Salmonella*	>96	100
	992.11^*g*^	Official Method	Biocontrol Assurance Gold EIA *Salmonella*	>79	100
	989.14^*h*^	Official Method	Tecra *Salmonella *VIA	*>70*	>78
	--	--	Bacteriological Analytical Method Chapter 5 ^23^	--	--

*a *Reference [25] (Sensitivity and specificity reported in liquid milk and apple cider)

*b *Reference [26] (Sensitivity and specificity reported in liquid milk and apple cider)

*c *Reference [27] (Sensitivity and specificity reported in apple cider)

*d *Reference [28] (Sensitivity and specificity reported in liquid milk)

*e *Reference [29] (Sensitivity and specificity reported in milk chocolate)

*f *Reference [27] (Sensitivity and specificity reported in orange juice, lettuce rinse, sprout rinse, chicken rinse)

*g *Reference [30] (Sensitivity and specificity reported in apple cider)

*h *Reference [31] (Sensitivity and specificity reported in pepper, soy flour, nonfat dry milk, raw ground poultry, chocolate)

## Results

### Hold-and-release testing

Hold and release testing results representing a total of 3216 samples were obtained from 13 growers. The majority of growers collected at least some of their samples as a composite from several drums. A few growers collected all samples from a single drum. Twenty five of the samples provided were derived from an unknown number of drums. None of these 25 samples resulted in presumptive positives and they are omitted from further reporting of the data, thus leaving 3191 samples from 6839 drums. The distribution of samples based on the number of drums composited is presented in Table 2.

**Table 2:** Distribution of number of drums composited into each sample for pathogen testing.

Composite of:	No. samples	Percent ^*a*^
Single drum	1805	57
2–4 drums	1171	37
5–7 drums	166	5.2
8–19 drums	49	1.5

^*a *^Expressed as a percent of the 3191 samples for which the number of drums was known.

Whereas all growers produced broccoli sprouts, many of the composite samples taken included samples from non-broccoli sprouts (alfalfa in most cases). Presumptive detection of *Salmonella *spp. or *E. coli *O157:H7 occurred in 24 of 3191 samples (0.75%) from a total of 6839 drums. As described in the FDA Guidance [16], the detections were considered presumptive and in general, the manufacturers claims for sensitivity of these tests ranged from 70% to 100% and the specificity ranged from 78% to 100% (Table 1). The majority of the tests are reported to have a rate of false positive responses below 2%. In most cases of presumptive positives, the water sample was then reanalyzed. In two of these cases, a follow-up analysis was again positive for *Salmonella *spp., and the sprouts were destroyed. In a single case a presumptive detection of *E. coli *O157:H7 was assumed by the grower to be real without re-testing; the sprouts were destroyed. None of these 3 samples included broccoli sprouts. Additional prophylactic measures were taken as outlined in reference [16]. Sprouts were held at the growing facilities until microbial testing results were confirmed. There were no instances in which contaminated sprouts were released for distribution.

In order to determine whether detection of presumptive positives was affected by the number of drums pooled for assay, the 24 presumptive positive samples have been examined, relative to the number of drums sampled and pooled in a single assay (Table 3). Assuming that a presumptive positive was caused by a single drum, the frequency of occasions when a presumptive contamination was observed was calculated for each sample class. It thus appears that there is no loss of sensitivity when a single sample represents as many as seven drums although the number of presumptive positives observed in this review was limited. Based upon the Mann-Kendall Trend Test for Small Sample Size [24], there is no trend for the percent of presumptive positives per drum (*p *≤ 0.01), between one (single) and 7 (pooled) drums, at the 95% confidence level.

**Table 3:** Presumptive positive samples grouped by composite number of drums represented by the water sample tested.

No. of drums	No. of presumptive positives	No. of samples	Total No. of drums	Presumptive positives per drum (%)
1	10	1805	1805	0.554
2	4	259	518	0.772
3	4	558	1674	0.239
4	2	354	1416	0.141
5	2	69	345	0.580
6	0	63	378	0.000
7	2	34	238	0.840
8–19	0	49	465	0.000
Overall	24	3191	6839	0.351

## Discussion

HACCP-based microbial hold-and-release testing, conducted in concert with strict seed and facility sanitation procedures by 13 U.S. broccoli sprout growers (representing tests of 6839 drums of sprouts or about 5 million consumer packages of fresh green sprouts) has resulted in the successful identification and elimination of hazardous microbial contamination when and where it existed. Less than half a percent of the samples tested gave an initial positive test for *E. coli *O157:H7 or *Salmonella *spp. When re-tested, only 10% of these (3 drums out of 6839), were positive for the presence of these organisms. By using a "test-and-re-test" protocol, growers were able to minimize crop destruction. By pooling drums for testing, they were also able to reduce testing costs which now represent a substantial portion of the costs associated with sprout growing. The test-and-hold scheme described herein allowed those few batches of contaminated sprouts to be found prior to packaging and shipping. These events were isolated, and only safe sprouts entered the food supply.

With proper attention to growing conditions and testing procedures, the advantages of fresh green sprouts can be safely realized by those who choose to eat sprouts as part of a healthy diet.

## Competing interests

One of the authors (JWF), as well as Johns Hopkins University, own stock in Brassica Protection Products (BPP), a company whose mission is to develop chemoprotective food products and which sells broccoli sprouts. JWF is a co-founder, and an unpaid scientific consultant to BPP, and his stock is subject to certain restrictions under University policy. The terms of this arrangement are being managed by Johns Hopkins University in accordance with its conflict of interest policies. One of the authors (PJO) is employed by a company that provides independent audit and quality assurance services to BPP.

## Authors' contributions

JWF participated in the design and coordination of the study and drafted the manuscript. PJO collected and analyzed the data. FHD advised on appropriate study design and participated in development of the manuscript. All authors read and approved the final manuscript.
